# Supplementary figures and images for: Transfer of endogenous small RNAs between branches of scions and rootstocks in grafted sweet cherry trees
Source: PLoS One. 2020 Jul 28;15(7):e0236376. doi: 10.1371/journal.pone.0236376 (PMC7386610; doi:10.1371/journal.pone.0236376)

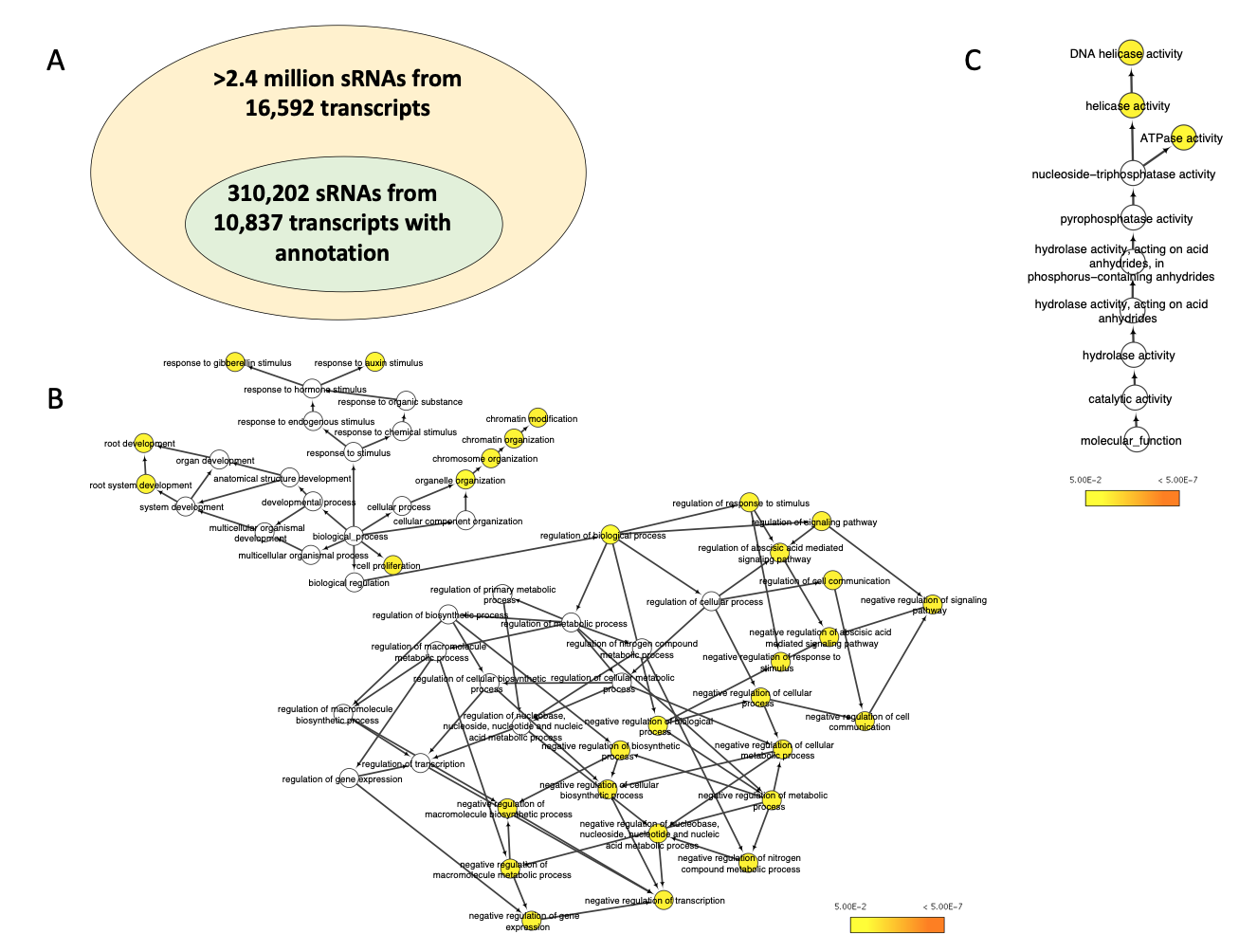

Supplement: S1 Fig — Summary of sRNAs (A) and gene networks of overrepresented sRNAs in bud tissues of sweet cherry scion 19 (Scion19) ‘Emperor Francis’ grafted on a ‘Gisela 6’ rootstock (RS19). The ontology file of GO_FULL in BiNGO and A. thaliana annotation were used as the references to identify overexpressed GO terms (P < 0.05). Bubble color indicates the P-value. Overrepresented sRNAs in “Biological process” (B) and “Molecular function” (C). No overrepresented GO terms are present in “Cellular component”. (TIFF) [file pone.0236376.s001.tiff]
