# Supplementary material for: Transfer of endogenous small RNAs between branches of scions and rootstocks in grafted sweet cherry trees
Source: PLoS One. 2020 Jul 28;15(7):e0236376. doi: 10.1371/journal.pone.0236376 (PMC7386610; doi:10.1371/journal.pone.0236376)
Supplement: Attachment — Submitted filename: Response_v2_20200630.docx [file pone.0236376.s007.docx]

Please use the space provided to explain your answers to the questions above. You may also include additional comments for the author, including concerns about dual publication, research ethics, or publication ethics. (Please upload your review as an attachment if it exceeds 20,000 characters)

Reviewer #3: In this paper, the small RNA differences among different scions and rootstocks in grafted sweet cherry trees were analyzed, and a batch of endogenous sRNAs for potential long-distance scion-to-rootstock transfer was screened. This study provided references for sRNA function research in the biological processes of the recipient plants.
Overall, the author has replied the questions raised by Reviewer #1 and Reviewer #2, and the manuscript has been modified accordingly. However, due to the understanding of the role of the long-distance transported sRNAs in sweet cherry was all based on sRNAs profiles, It is suggested that the information about sequencing depth and quality of the original data should be provided in detail to prove the reliability of the relevant conclusions.

**Response:** Thanks for your suggestion. We added Table 1 (the summary of small RNA sequencing reads) and mentioned the read information in the manuscript as follows:

“Overall, a total of 63 million 50 bp single-end reads were generated, where >96% were with Phred qualities equal to or greater than 30 (Table 1).”
